# Reviewer acknowledgement 2013

**DOI:** 10.1186/1471-2172-15-7

**Published:** 2014-03-13

**Authors:** Helena C Flemyng

**Affiliations:** 1 BioMed Central, Floor 6, 236 Gray’s Inn Road, London WC1X 8HB, UK

## Abstract

**Contributing reviewers:**

The editors of BMC Immunology would like to thank all our reviewers who have contributed to the journal in Volume 14 (2013).

## 

Jakub Abramson

Israel

Joeri Aerts

Belgium

Neil Aggarwal

United States of America

Omid Akbari

United States of America

Rebeca Alonso-Arias

Spain

Susana Alvarez

Argentina

Yassine Amrani

United Kingdom

Anna Andersson

Sweden

Amilcar Arenal

Brazil

Ali Ashkar

Canada

Paul Ashwood

United States of America

Deshratn Asthana

United States of America

Athan Baillet

France

Anand Balasubramani

United States of America

Alberto Baldini

Italy

Taravat Bamdad

Iran

James Baraniuk

United States of America

Surinder Batra

United States of America

Elyse Bissonnette

Canada

Ulrich Blank

France

Russell Blaylock

United States of America

Antje Blumenthal

Australia

Maria Da Gloria Bonecini-Almeida

Brazil

Karina Bortoluci

Brazil

Marcelo Bozza

Brazil

Thorsten Brenner

Germany

Charles Brown

United States of America

Todd Brusko

United States of America

Peter Burbelo

United States of America

Anne Caignard

France

Martin Cannon

United States of America

Michael Carleton

United States of America

Beatriz Carreno

United States of America

Robert Chevalier

United States of America

Seok-Goo Cho

South Korea

George Cianciolo

United States of America

Stephen Cobbold

United Kingdom

David Cousins

United Kingdom

Julio Croda

Brazil

Mard Daëron

France

Hong Dai

United States of America

Gautam Damera

United States of America

Jayasri Das Sarma

India

Luis De La Maza

United States of America

Tom Decoursey

United States of America

Stephanie Dewitte-Orr

Canada

Umberto Dianzani

Italy

David Dockrell

United Kingdom

Taylor Doherty

United States of America

Harry Dolstra

Netherlands

Jeffrey Ebersole

United States of America

Bernd Echtenacher

Germany

Tatjana Eigenbrod

Germany

Abdallah Elkhal

United States of America

Anat Erdreich-Epstein

United States of America

Monica Facco

Italy

Chiara Fenoglio

Italy

Constantin Fesel

Portugal

Brian Fife

United States of America

Carlos Flores

Spain

Claudio Fozza

Italy

Adam Enver Frampton

United Kingdom

Alessandra Franco

United States of America

Jianing Fu

United States of America

Patricia Fulkerson

United States of America

Olivier Garraud

France

Paramita Ghosh

United States of America

Bernhard F Gibbs

United Kingdom

Sarah Gilbert

United Kingdom

Paul Goepfert

United States of America

Abdelilah Gounni

Canada

Veronika Grau

Germany

Jason Grayson

United States of America

Eduardo Gudo

Mozambique

Koji Hase

Japan

Catherine Hawrylowicz

United Kingdom

Helena Helmby

United Kingdom

Catharien Hilkens

United Kingdom

Sunil Hingorani

United States of America

Ikuo Hirono

Japan

Biliang Hu

United States of America

Shunji Ishihara

Japan

David Jayne

United Kingdom

Maria Jenmalm

Sweden

Nobuhiko Kamada

United States of America

Jia-Horng Kao

Taiwan

Susumu Katsuma

Japan

Can Kesmir

Netherlands

Sonja Kierstein

Austria

Ulf Klein

United States of America

Naoki Koide

Japan

Pascale Kropf

United Kingdom

Stefanie Kuerten

Germany

Jun Kunisawa

Japan

William Kwok

United States of America

Yu-Lung Lau

Comoros

Agnes Lehuen

France

Francesca Levi-Schaffer

Israel

James Li

Hong Kong

Min Li

China

Xiaokun Li

China

Peter Linsley

United States of America

Baoyu Liu

United States of America

Brian Long

United States of America

Enrico Lugli

Italy

Andreas Lux

Germany

Yoshinobu Maeda

Japan

Jacopo Mariotti

Italy

Per Marits

Sweden

Ben Marsland

Switzerland

María Martínez-Esparza

Spain

Mitsuru Matsumoto

Japan

Anne Kathrin Mausberg

Germany

Juan Manuel Mejia-Arangure

Mexico

Tuuli Metsvaht

Estonia

David Meya

Uganda

Gerd Meyer Zu Hörste

Germany

Aruna Mittal

India

Philippe Moingeon

France

Markus Moll

Sweden

Marcin Moniuszko

Poland

Laurence Morel

United States of America

Laura Morf

Switzerland

Eiichi Morii

Japan

James Moss

United States of America

Eric Mossel

United States of America

Dimitrios Mougiakakos

Germany

Victoriano Mulero

Spain

Michael Muller

Australia

David Mullins

United States of America

Kimihiko Nakatani

Japan

Maki Nakayama

United States of America

Alistair Noble

United Kingdom

Susanne Nylen

Sweden

Nobuo Okahashi

Japan

Ebru Olgun Erdemir

Turkey

Connor O'Meara

Australia

Eric Oswald

France

George Pappas-Gogos

Greece

Nikhat Parveen

United States of America

Marina Pasca Di Magliano

United States of America

Daniel Perry

United States of America

Luca Persani

Italy

Paul Pfeffer

United Kingdom

Kenneth Pollard

United States of America

Ilaria Puxeddu

Italy

Govindarajan Rajagopalan

United States of America

Roger Keith Reeves

United States of America

Alexandre Reis

Brazil

Eleanor Riley

United Kingdom

Fabricio Rios-Santos

Brazil

Chiara Romagnani

Germany

Lijun Rong

United States of America

Stefano Rossetti

United States of America

Andrew Rowley

United Kingdom

Nadine Rujeni

Rwanda

Matías Sáenz-Cuesta

Spain

Sampa Santra

United States of America

Ghanashyam Sarikonda

United States of America

Marion Scharpfenecker

Netherlands

Stephan Schlickeiser

Germany

Bianca Schneider

Germany

Christian Schönbach

Japan

Michael Seaman

United States of America

Mårten Segelmark

Sweden

Boualem Sendid

France

Yechiel Shai

Israel

Takeshi Shimosato

Japan

Yehuda Shoenfeld

Israel

Thomas Sisson

United States of America

Monica Fisher Smikle

Jamaica

Vsevolod Smolianov

Germany

Wenchao Song

United States of America

Cate Speake

United States of America

Lisa Spencer

United States of America

Andreas Spittler

Austria

Deogratius Ssemwanga

Uganda

Stephen St Jeor

United States of America

Simona Stager

Canada

Melanie Stumpf

United States of America

Chuan Su

China

Jie Sun

United States of America

Kei Takahashi

Japan

Beatriz Tavares Costa Carvalho

Brazil

Elma Tchilian

United Kingdom

Alberto Tedeschi

Italy

Wayne Thomas

Australia

Wolfgang Tillinger

Austria

Masanori Tohno

Japan

Giovanni Tonon

Italy

Tim Tree

United Kingdom

Marten Trendelenburg

Switzerland

Marita Troye-Blomberg

Sweden

Akihito Tsubota

Japan

Sung Hee Um

South Korea

Luc Van Kaer

United States of America

Audard Vincent

France

Mark Wallet

United States of America

Dapeng Wang

United States of America

Clemens Warnke

Germany

Clive Wasserfall

United States of America

Napoleon Waszkiewicz

Poland

Marc Wathelet

United States of America

Winfried S Wels

Germany

Thilo Welsch

Germany

Johnna Wesley

United States of America

Geert Wiegertjes

Netherlands

Heather Wilson

United Kingdom

Ola Winqvist

Sweden

Hao Wu

China

Aini Xie

United States of America

Zhou Xing

Canada

Mahesh Yadav

United States of America

Karina Yazdanbakhsh

United States of America

Li Ye

United States of America

Xiaozhou Ying

China

Sanhong Yu

United States of America

Yangsheng Yu

United States of America

Margit Zeher

Hungary

Andrea Zelmer

United Kingdom

Ming Zeng

United States of America

Juan Carlos Zuniga-Pflucker

Canada

